# Local cryotherapy improves adjuvant-induced arthritis through down-regulation of IL-6 / IL-17 pathway but independently of TNFα

**DOI:** 10.1371/journal.pone.0178668

**Published:** 2017-07-31

**Authors:** Xavier Guillot, Hélène Martin, Stéphanie Seguin-Py, Katy Maguin-Gaté, Johnny Moretto, Perle Totoson, Daniel Wendling, Céline Demougeot, Nicolas Tordi

**Affiliations:** 1 PEPITE EA4267, FHU INCREASE, Univ. Bourgogne Franche-Comté, Besançon, France; 2 Service de Rhumatologie, CHRU Besançon, France; 3 INSERM CIC 1431, FHU INCREASE, Besançon Cedex, France; 4 EA 4266, Univ.Bourgogne Franche-Comté, Besançon, France; Northwestern University Feinberg School of Medicine, UNITED STATES

## Abstract

**Objectives:**

Local cryotherapy is widely and empirically used in the adjuvant setting in rheumatoid arthritis treatment, however its own therapeutic and anti-inflammatory effects are poorly characterized. We aimed to evaluate the effects of local cryotherapy on local and systemic inflammation in Adjuvant-induced arthritis, a murine model of rheumatoid arthritis.

**Methods:**

The effects of mild hypothermia (30°C for 2 hours) on cytokine protein levels (Multiplex/ELISA) were evaluated *in vitro* in cultured rat adjuvant-induced arthritis patellae. *In vivo*, local cryotherapy was applied twice a day for 14 days in arthritic rats (ice: n = 10, cold gas: n = 9, non-treated: n = 10). At day 24 after the induction of arthritis, cytokine expression levels were measured in grinded hind paws (Q-RT-PCR) and in the plasma (Multiplex/ELISA).

**Results:**

*In vitro*, punctual mild hypothermia down-regulated IL-6 protein expression. *In vivo*, ice showed a better efficacy profile on the arthritis score and joint swelling and was better tolerated, while cold gas induced a biphasic response profile with initial, transient arthritis worsening. Local cryotherapy also exerted local and systemic anti-inflammatory effects, both at the gene and the protein levels: IL-6, IL-17A and IL-1β gene expression levels were significantly down-regulated in hind paws. Both techniques decreased plasma IL-17A while ice decreased plasma IL-6 protein levels. By contrast, we observed no effect on local/systemic TNF-α pathway.

**Conclusions:**

We demonstrated for the first time that sub-chronically applied local cryotherapy (ice and cold gas) is an effective and well-tolerated treatment in adjuvant-induced arthritis. Furthermore, we provided novel insights into the cytokine pathways involved in Local cryotherapy’s local and systemic anti-inflammatory effects, which were mainly IL-6/IL-17A-driven and TNF-α independent in this model.

## Introduction

Rheumatoid arthritis (RA) is an auto-immune disease in which synovial inflammation causes pain, joint destruction, disability. In the inflamed joint, macrophages, T and B lymphocytes, synoviocytes secrete pro-inflammatory cytokines and induce enzymes involved in synovial inflammation and cartilage degradation (Cyclo-oxygenase-2 (COX-2), inducible-NO synthase (i-NOS), metalloproteinases) under the direct control of pivotal transcription factors such as Nuclear Factor-kappa B (NF-kB) [1]. Local and systemic inflammation are driven by specific CD4+ T lymphocyte subsets and cytokine profiles resulting in a complex pro-inflammatory cytokine network [2]. RA was first considered as a T-helper 1 (Th1)-driven disease, mainly depending on IFN-ɤ/TNF-α pathway and Th1/Th2 balance [3]. More recently, IL-6 has been shown to induce Th-17 differentiation and to decrease regulatory-T cell (Treg) expansion in murine models of RA, promoting a pro-inflammatory immune response profile [4–6]. These IL-6/IL-17 axis and Th-17/Treg balance have emerged as a major pathogenic pathway in RA [7], through widely TNF-independent mechanisms [8,9]. Consistent with these hypotheses, both TNF-α and IL-6 blockades are efficient therapeutic strategies in RA [10]. Despite these highly efficient targeted biologic treatments, many arthritic patients still need adjunct anti-inflammatory drugs such as corticosteroids or non-steroidal anti-inflammatory drugs (NSAIDs), known to be iatrogenic and to increase morbidity and mortality [11]. Therefore, the use of adjunct anti-inflammatory treatments with a better risk-benefit balance could be very helpful in RA.

Cryotherapy is empirically used in the adjuvant setting in RA treatment, with a very good tolerance profile as compared to corticosteroids or NSAIDs, but protocols lack standardization. A recent systematic review of literature, reported that local (ice, cold gas) as well as whole-body cryotherapy (WBC) applied 2 times a day for 7 to 15 days (14–20 applications) significantly decreased pain Visual Analogic Scale (VAS) and Disease Activity Score assessed on 28 joints (DAS28) in 257 RA patients [12]. The majority of the few available studies regarding cryotherapy in RA patients suffer from many limitations related to the lack of assessment of associated physical and pharmacological treatments, a high level of polymedication, the lack of control group, small sample sizes, the heterogeneity in cryotherapy protocols (physical agents, temperature, duration, periodicity, indications). Moreover, it would be impossible to conceive a placebo mimicking cryotherapy. In this context, the use of animal models of arthritis is a benchmark choice to address the own effects of long-term cryotherapy in RA. Previous studies in many physiological and pathological conditions identified cryotherapy related analgesic [13], antiphlogistic, myorelaxing [14,15], vasoconstrictive [16], anti-inflammatory [17], enzyme-blocking [18] and anti-oxidative [19] effects. These effects were related to NF-kB-dependent IL-1β, IL-6 and TNF-α gene transcription inhibition [20], temperature-dependent enzyme metabolism blockade [21]. However, whether these mechanisms are involved in cryotherapy-treated RA is not known.

In the present study, we investigated the effect of a sub-chronic treatment with local cryotherapy (LC) in the widely-used rat model of adjuvant-induced arthritis (AIA). Two modalities of cryotherapy were compared: local ice and cold spray application. After 14-days of daily treatment, the effect of cryotherapy on hind paw cytokines gene expression, plasma cytokine levels, hind paw diameter and arthritis scores were assessed. To determine the effect of temperature by itself, cytokines levels were also measured in vitro in a cultured patellar explant model after exposure to a mild hypothermia (30°C).

## Materials and methods

### Animals

Six-week old male Lewis rats (n = 30 for in vivo experiments, n = 10 for in vitro experiments) were purchased from Janvier (Le Genest Saint Isle, France). Animals were kept under a 12h-12h light: dark cycle and allowed free access to food and water. The experimental procedures were approved by the local committee for ethics in animal experimentation (n° 2012–019) of Franche-Comté University (Besançon, France), and complied with the Guide for the Care and Use of Laboratory Animal published by the US National Institutes of Health (NIH publication No. 85–23, revised 2011).

### Induction and clinical evaluation of the arthritis model

Adjuvant arthritis was induced by a single intradermal injection at the base of the tail of 120 μL of 1 mg of heat-killed Mycobacterium butyricum (Difco^®^, Detroit, MI) suspended in 0.1 ml of mineral oil (Freund’s incomplete adjuvant (Difco^®^, Detroit, MI)). The AIA model is characterized by rapid onset and progression of a robust and easily measurable polyarthritis, clinically characterized by severe erythema and diffuse soft swelling with complete ankylosis and malformations in the paws, reduced locomotor activity, frequently associated to ears and tail inflammation, weight loss, anorexia and diarrhea [22]. From the beginning of arthritis, rats were manipulated with extreme precaution in order to minimize pain. When necessary, the painful rats were isolated from the other animals and placed in a separate cage. Furthermore, as arthritis severely impaired the rat mobility during the inflammatory phase, food was placed directly on the litter at the bottom of the cage. Rats were weighed and monitored 7 days per week for ankle diameter and clinical signs of arthritis. These parameters are part the core set of variables which are usually collected for the pre-clinical evaluation of RA in murine models of arthritis according to the official international guidelines [23]. The scoring system was employed as follows [24]: arthritis of one finger scores 0.1, weak and moderate arthritis of one big joint (ankle or wrist) scores 0.5 and, intense arthritis of one big joint scores 1. Tarsus and ankle were considered as the same joint. Sum of joints scores of 4 limbs leads to an arthritic score of maximum 6 to each rat. The ankle diameter was measured with a digital caliper (Vernier Stainless^®^, China). A group of non-arthritic age-matched rats was used as controls and received saline at the base of the tail. Weight loss is one of the first arthritis clinical symptoms. However, for rats exhibiting more than 20% of weight loss, humane endpoints will be evaluated according to the criteria and scoring proposed by Lloyd and Wolfensohn in 1998 [25], taking into account physical appearance, behavior, hydratation and clinical respiratory symptoms of the rat. Depending on the degree of suffering of the animal, rats exhibiting severe suffering will be euthanized by intra-peritoneal injection of a lethal dose of sodium pentobarbital CEVA (200 mg/kg, ip). In our experiments, as no rat showed any 20% or more weight loss, the limit points were not evaluated and no rat was euthanized prior to the planned end of the experiments. One rat died from a cardiac arrest prior to the experimental endpoint, during the very first application of cold gas spray. Therefore, only 9 AIA rats were considered in the cold gas treatment group.

At the day of the first inflammatory symptoms (i.e. at day 10–11 post-immunization) AIA rats were randomized in 3 groups. One group received ice application (n = 10), one group received cold spray application (n = 10), one group was untreated (n = 10). Local cryotherapy was applied to both hind paws twice a day with a 8-hour interval (at 9 A.M and 5 P.M) for 14 consecutive days. Room and skin temperature were monitored using MLT409/A^®^ Skin Temperature Probe (ML309 transducer Thermistor Pod—AD Instruments). Skin temperature was measured just after each cold application on hind paws (on both tarsae and ankles in alternating order of measurement). As background, some authors reported that in the Wistar rat monoarticular AIA model, the mean skin temperature measured by using a thermal camera, was 35.7 ± 0.68°C on the right arthritic hind paws (N = 15), while the mean value was 34.72 ± 0.29°C on the corresponding contralateral non-arthritic left hind paws (N = 15) [26]. The mean room temperature over 14 days (mornings and afternoons) was slightly lower in ice-treated group compared to cold gas spray–treated animals (23.14°C±0.17 versus 23.59±0.14°C; p = 0.049). For ice application, the bottom of 5 cages (2 rats per cage separated by ice chips) was lined with 50 mL ice pops (Yéti^®^, Yetigel, Avignon, France) previously frozen at -26°C. Rats were installed in the ice-lined cages for 30 minutes. Rats were placed in the cages and removed 30 minutes later at a 5 minute-interval in an alternating order. As concerns gas LC, rats were treated twice a day for 14 consecutive days using cold sprays (Ice Spray, Ghiaccio Spray^®^, Artsana Group, Italy. CE 0546). Cold gas was pulverized on each hind paw at a distance of 25 cm (Nine 5 second-applications alternated with 10 second breaks during 2 minutes for each hind paw). The orders of treatment for rats and paw sides were also alternated. Cryotherapy-treated rats were killed the day after the last cold application.

### Tissue collection

Twenty-four days after treatment initiation, rats were anaesthetized with pentobarbital (60 mg/kg, i.p.). Blood was withdrawn from the abdominal artery and centrifuged to obtain plasma, divided into aliquots and stored at -80°C until analysis. Hind paws were dissected just above ankles, then prepared as described in the Q-RT-PCR section.

### Cultured patellae explants

Separated group of AIA (n = 10) and control Lewis rats (n = 10) were used to assess the anti-inflammatory effects of one local cold application in synovial tissues. At day 24 post-immunization, after anaesthesia with pentobarbital (60 mg/kg, i.p.), both patellae were dissected according to the method described by Lubberts et al. [27–30]. Patellae were then cultured in RPMI 1640 medium (200 microliters per patella) mixed with mBSA (0.1%), gentamycin (50 micrograms per mL) and L-Glutamin (2 mM) at 37°C (left patellae) versus 30°C (right patellae) for 2 hours, in order to reproduce synovial temperature conditions in local ice-treated arthritic knees in humans (tissue mild hypothermia) [31]. Culture supernatants were then harvested. The effect of hypothermia on cell viability was assessed by measuring ATP levels in patellae by luminescence. The results showed that hypothermia did not affect cell viability of the explant [S1 Fig]. Supernatants were aliquoted and stored at -80°C until analysis.

### Q-RT-PCR

In order to assess long-term local cryotherapy anti-inflammatory effects, hind paws from AIA rats treated by cryotherapy and non-treated controls were cut just above the ankles. Skin and claws were dissected, then paws were directly frozen in liquid nitrogen then stored at -80°C. Frozen paws were then used for quantitative real-time PCR analysis as described in [32–38] Frozen paws were then finely grinded in a mortar, mRNA were extracted using Qiagen^®^ mRNA extraction kit, then Q-RT-PCR were performed using Biorad^®^ cDNA and SybrGreen kits. TNF-α, IL-1β, IL-6, IL-17A gene expression were quantified in paws (Primer sequences: Actin: sense TATCGGCAATGACGCGTTCC–antisense GCCTGGGTACATGGTGGTG; GADPH: sense GGGCATCCTGGGCTACACTG-antisense GAGGTCCACCACCCTGTTGC; TNF-α: sense CCAATCTGTGTCCTTCTAA-antisense TTCTGAGCATCGTAGTTG; IL-6: sense GACCAAGACCATCCAACT-antisense TAGGTTTGCCGAGTAGAC; IL-1β: sense CCTGCAGCTGGAGAGTGTGGAT-antisense TGCTCTGCTTGAGAGGTGCTG; IL-17A: sense AACAGAGACCTGAGGCTA–antisense TCCATATCACTTGCTGAGATT).

### Western blot

Frozen powder of paws obtained as described in the Q-RT-PCR section were homogenized in a lysis buffer (phosphate-buffered saline with EDTA 2 mM, EGTA 2 mM, PMSF 1 mM, aprotinin 2 mM, leupeptin 2 mM, SDS 1%), sonicated for 2 min and centrifuged at 12 000 g for 10 min at 4°C. The supernatants were used for protein quantification using a commercially available bicinchoninic acid protein determination kit with bovine serum albumin as a standard. Pooled samples were then prepared by mixing supernatants from all rats of a given animal group, the same protein quantity being provided by each rat. Proteins (40 μg) were separated on 15% polyacrylamide gels and transferred to PVDF membranes for 1 h in Tris-Glycine buffer. Membranes were saturated in 0.1% TBS-Tween 20 and 5% nonfat milk for 1 h and then incubated overnight at 4°C with a rabbit anti-TNF-α (1/1000, Abcam ab6671), a mouse anti-IL6 (1/1000, Abcam ab9324), a rabbit anti-IL-1β (1/1000, Abcam ab9722), a rabbit anti-IL17 (1/5000, Abcam ab79056) antibodies and a mouse anti-actin (1/20000, Millipore mab1501R) antibody as a control. Membranes were washed 3 times with TBS-Tween 20 0.1%, incubated for 1h at room temperature with secondary anti-rabbit or anti-mouse HRP conjugate antibodies (1/10000 in 0.1% TBS-Tween 20^®^, Jackson ImmunoResearch). The membrane was washed 3 times with TBS-0.1% Tween 20^®^ and then incubated with ECL revelation buffer (ECL+^®^, GE Healthcare, France) and chemiluminescence was monitored using a ChemiDocXRS+^®^ (Biorad).

### Plasma and supernatant cytokine levels

Levels of TNF-α, IL-17A and IL-1β levels were measured using Milliplex magnetic bead panel kits (eBioscience, Vienna, Austria) that were analyzed using a Luminex MAGPIX system (Luminex Corporation; Houston, TX) and Milliplex Analyst software (Millipore; St. Charles, MO). The limits of detection provided by the manufacturer for IL-17A, IL-1β and TNFα were 3.32 pg/mL, 13 pg/mL and 3.78 pg/mL respectively. IL-6 levels were measured using ELISA technique (rat IL6 platinum ELISA, BMS625^®^, eBioscience, Vienna, Austria—detection limit: 12 pg/ml).

### Statistical analysis

Values are presented as means ± SEM. In order to compare the evolution of arthritic scores, ankle diameters, weight gain (%) and cytokine levels in hind paws and plasma in ice, cold gas or non-treated rats, non-parametric Mann-whitney tests and 2-way repeated measures ANOVA were performed. Bonferroni post-tests were performed in order to compare daily values between groups. As for patella culture assays, paired Wilcoxon tests were used. The type I error coefficient was set at 0.05. Pearson’s coefficients were used. Statistical analyses were performed using R and Graphpad^®^ softwares.

## Results

### Effects of mild hypothermia on cytokine protein expression in cultured AIA patellar explants in vitro

**Fig 1:**
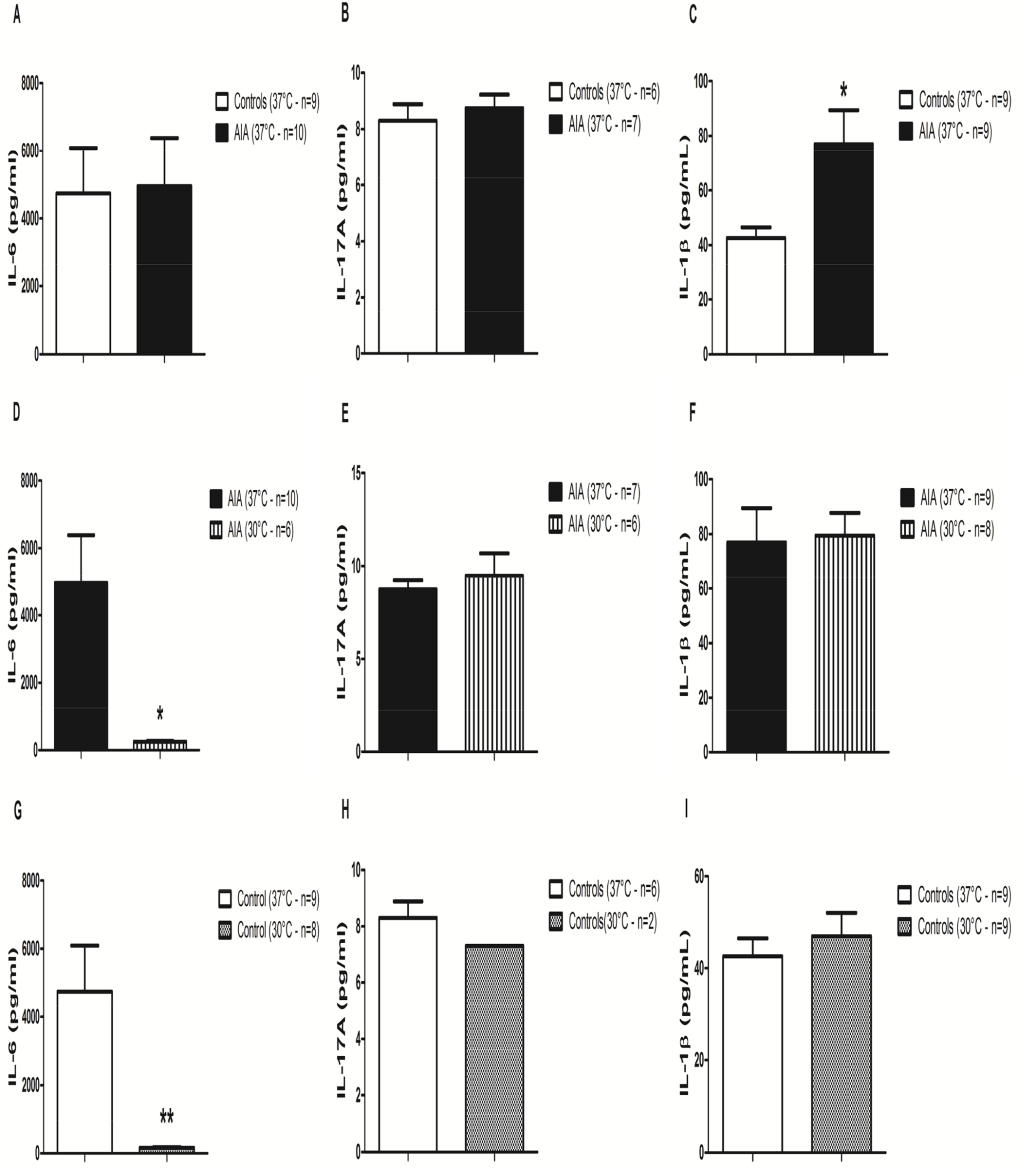
Cytokine levels in patellar explant culture supernatants from control and AIA rats. Both patellae from AIA (n = 10) and non-AIA (n = 10) rats were cultured at 30°C (right patellae) versus 37°C (left patellae) for 2 hours. IL-6 (**A, D, G**), IL-17A (**B, E, H**) and IL-1β (**C, F, I**) levels were measured in culture supernatants. Results are expressed as means ± SEM. Paired Wilcoxon tests were used. * p<0.05, ** p<0.01, ***p<0.001.

At 37°C, IL-1β levels were significantly higher in arthritic AIA compared to control rats (p = 0.05 –Fig 1C). Conversely, no difference was measured for IL-6 (Fig 1A) nor IL-17A (Fig 1B) levels.

In AIA, as compared to 37°C, hypothermia significantly decreased IL-6 levels (p<0.05 –Fig 1D) but not IL-17A (Fig 1E) nor IL-1β (Fig 1F) protein levels. The same results were obtained in control rats (Fig 1G to 1I). TNF-α protein was not detectable in any culture supernatant, irrespective of the temperature and the arthritic status.

### Effects of local cryotherapy in AIA rats: Tolerance and skin temperature

**Fig 2:**
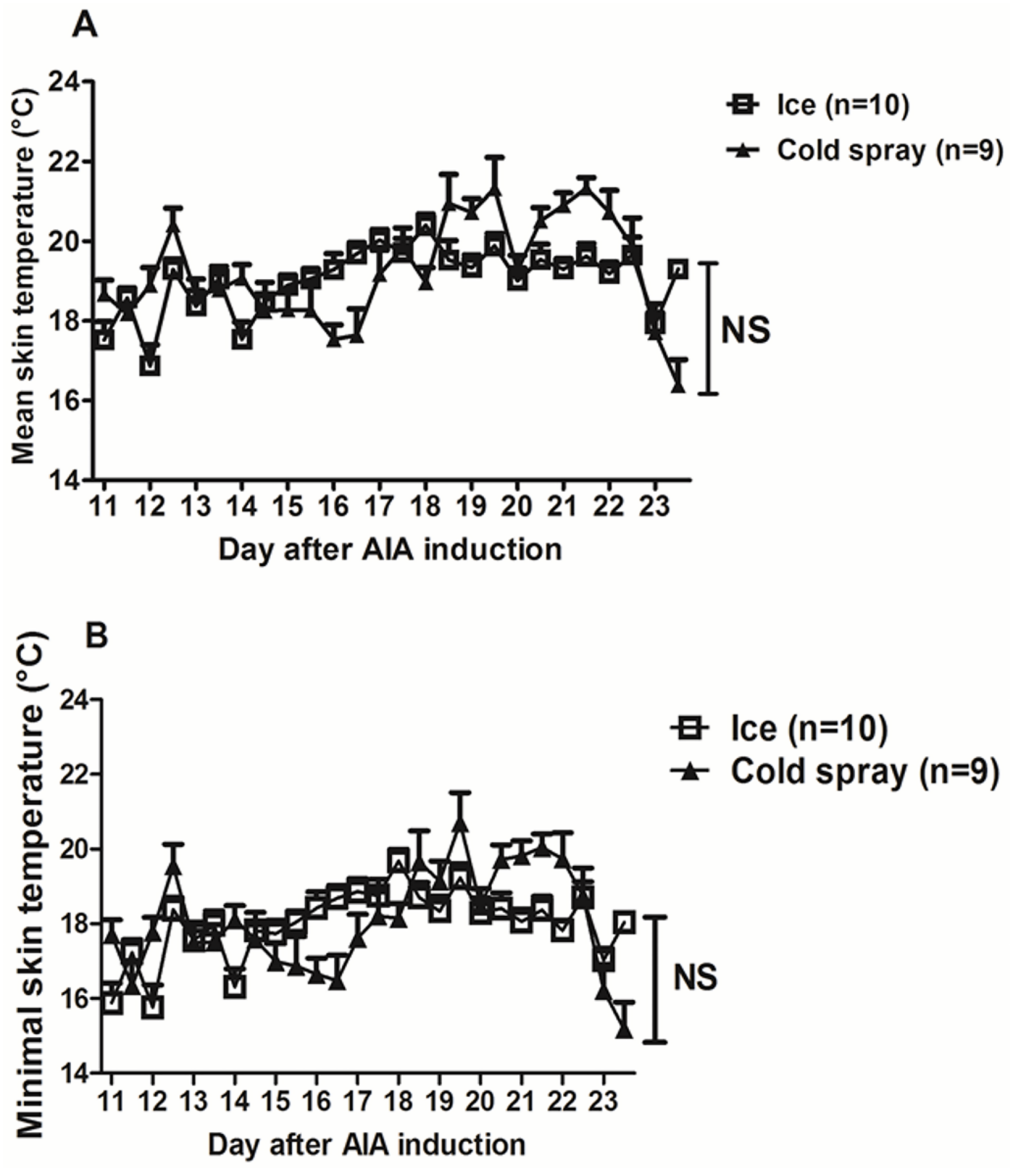
Skin temperature after cold application. Skin temperature was measured on hind paws (on both ankles and tarsae) just after each cold application using a temperature probe. The mean temperature (**A**) as well as the minimal temperature (**B**) on the 4 joints were calculated for each treated AIA rat. Results are expressed as means ± SEM (n = 9–10 rats). 2-way ANOVAs with Bonferroni post-tests were used. NS: non significant.

To determine whether the anti-inflammatory effects of hypothermia observed in vitro might translate into clinical benefits in vivo, AIA rats were treated for 14 days with local cryotherapy (ice or cold gas) twice a day. Cryotherapy was globally well-tolerated except for one rat who died from cardiac arrest during the first cold gas spray application. Cold gas-treated animals exhibited macroscopic signs of hind paw skin inflammation after one week under treatment. The mean body weight of AIA rats was unaffected by the treatment. At day 24, body weights (g) were 207 ± 3 in AIA, 211 ± 2 in ice-treated AIA rats (AIA-I) and 210 ± 3 in cold gas-treated AIA rats (AIA-CG) (NS). The mean skin temperature on treated ankles and tarsae over 14 days of treatment (mornings and afternoons) was 18.97 ± 0.8°C (mean ± SD) in ice-treated AIA rats and 19.17 ± 1.34°C in cold gas treated rats. The minimal temperature measured on the 4 sites over 14 days was 17.9 ± 0.92°C in AIA-I and 18 ± 1.38°C in AIA-CG. Mean and minimal skin temperatures didn’t differ between ice- and cold gas spray-treated arthritic rats throughout the 14 days of treatment (2-way ANOVA on repeated measures—Fig 2).

### Effects of local cryotherapy on clinical arthritis

**Fig 3:**
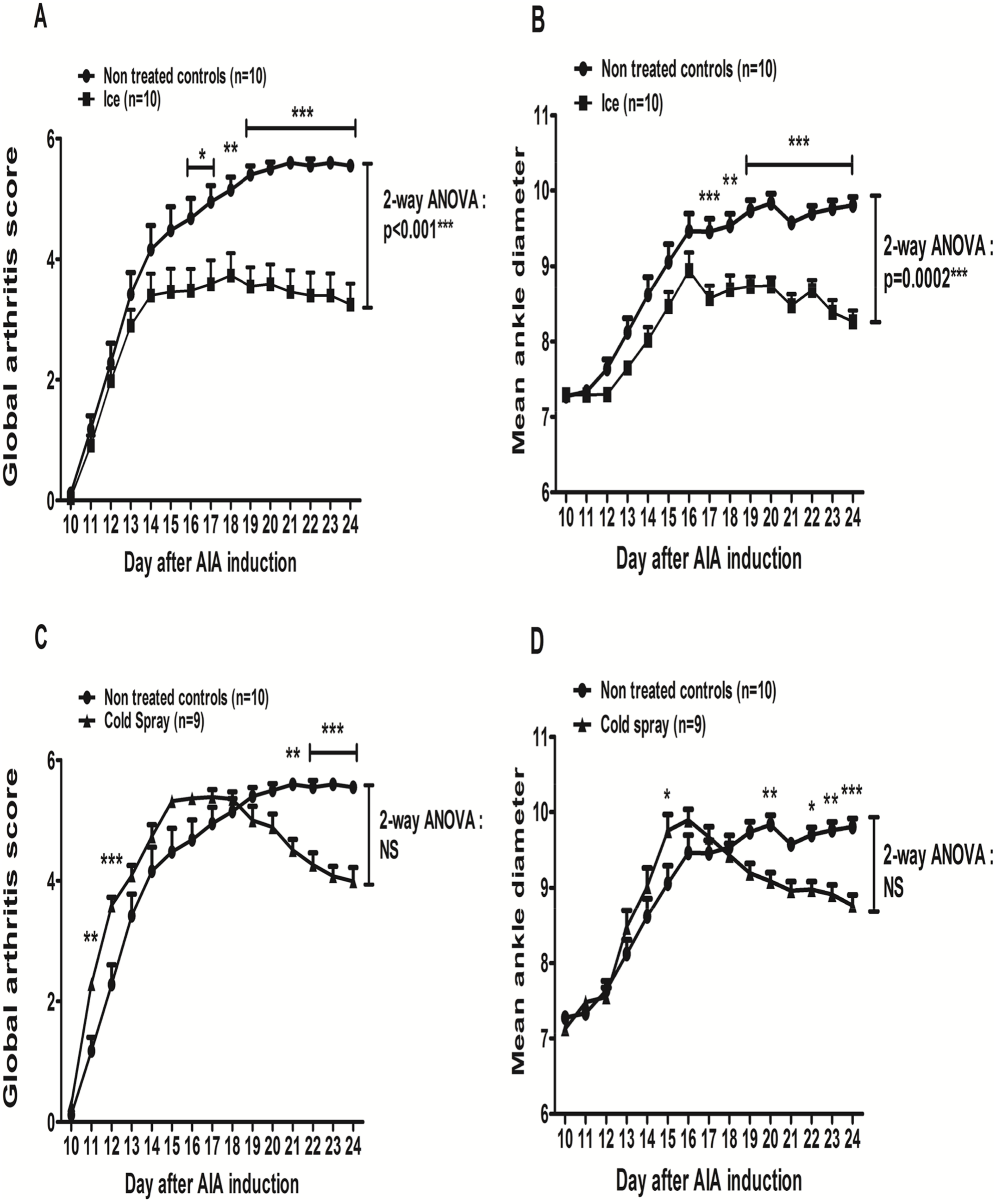
Effect of cryotherapy on the time-course of arthritis. AIA rats were treated or not (control) either by being placed into cages lined with ice pops for 30 minutes (n = 10) or by intermittent cold gas spray applications on hind paws for 2 minutes (n = 9), twice a day for 14 consecutive days from day 11 to day 24 post-immunization. Mean arthritis scores (**A, C**) and mean ankle diameter (**B, D**) were plotted over time after AIA induction. Results are expressed as means ± SEM (n = 9-10/group). 2-way ANOVAs on the whole 14 day treatment period were used and Bonferroni post-tests were performed at each evaluation time. ***p<0.001, **p<0.1, *p<0.05.

The first clinical signs of arthritis (paw edema, erythema, stiffness) appeared between day 11 and day 14 after immunization. From day 11 onwards, AIA rats experienced disease progression into a polyarthritis, characterized by severe erythema and diffuse soft swelling with complete ankylosis and malformations in the paws, reduced locomotor activity, frequently associated with ear and tail inflammation. In AIA, the arthritis score increased from day 11 until its maximum at day 21 (5.6 ± 0.3) and plateaued until day 24 (5.6 ± 0.3 at day 24 –Fig 3A and 3C). The time-course of the hind paw diameters was superimposable to that of arthritis score (Fig 3B and 3D). As compared to AIA, when considering the whole 14 day-treatment period, local ice cryotherapy significantly decreased arthritis score (p = 0.001—Fig 3A) and mean ankle diameter (p<0.001—Fig 3B) whereas the difference was not significant for cold spray therapy concerning the arthritis score (Fig 3C) and for the mean ankle diameter (Fig 3D). Actually, cold spray treatment induced a biphasic response (Fig 3C and 3D): it first aggravated the arthritis score at days 11–12 and then reduced the score at days 21–24 (Fig 3C).

Thus, even though the time-course was different, at the end at the treatment (day 24), both treatment significantly reduced arthritis score (- 41%, p<10^−4^ for ice and– 28%, p<0.001 for cold spray) and mean ankle diameters (-15.7%, p<0.0001 for ice and -10.6%, p = 0.0003 for cold spray) as compared to AIA. However, at day 24, mean arthritis scores tended to be lower in ice-treated compared to cold gas-treated rats (3.25 ± 0.35 versus 3.99 ± 0.23, NS) and mean ankle diameters were significantly lower in ice-treated rats (8.26 ± 0.15 mm) compared to cold gas (8.76 ± 0.14 mm, p<0.05). The time-course of the arthritis score on hind paws in each treatment group was superimposable to the time-course of the global arthritis score **[**S2 Fig].

### Effects of local cryotherapy on cytokine gene transcription in AIA hind paws

**Fig 4:**
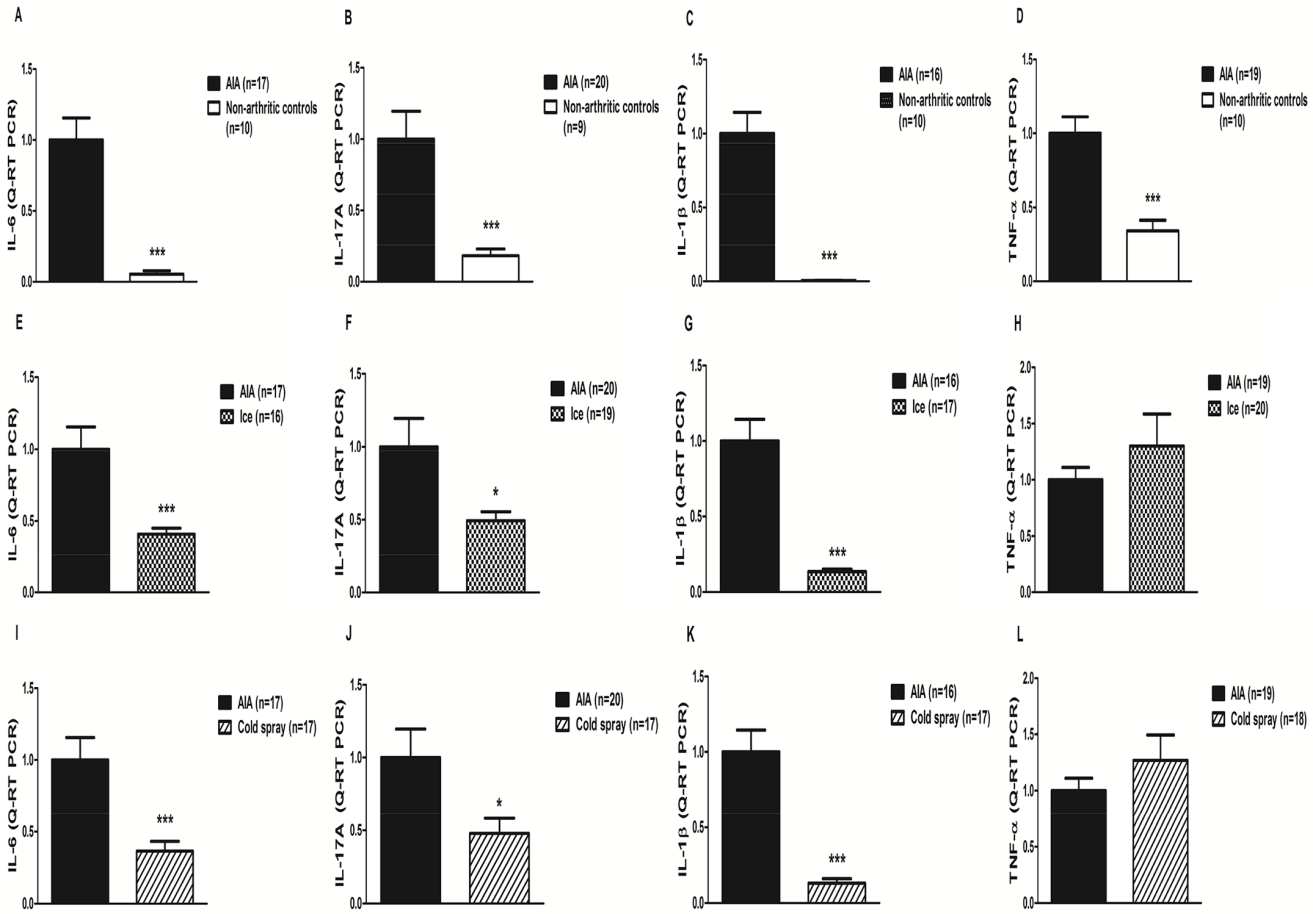
Mean cytokine levels in treatment groups (Q-RT-PCR on grinded hind paws). AIA rats were treated or not (control) either by being placed into cages lined with ice pops for 30 minutes (n = 10) or by intermittent cold gas spray applications on hind paws for 2 minutes (n = 9), twice a day for 14 consecutive days from day 11 to day 24 post-immunization. At day 24, Q-RT-PCR analyses of IL-6 (**A, E, I**), IL-17A (**B, F, J**), IL-1β (**C, G, K**) and TNF-α (**D, H, L**) gene expression were performed. Results are expressed as means ± SEM (n = 9-10/group). Mann-Whitney tests were used. ***p<0.001, *p<0.05.

To determine whether the clinical effect of 14 day-local cryotherapy was associated with local effects on cytokines, we measured cytokines gene expression in AIA groups at the end of the treatment period (day 24). As compared to control rats, AIA exhibited significantly higher IL-6, IL-17A, IL-1β and TNF-α gene expression (Fig 4A to 4D). Both ice and cold gas significantly decreased IL-6 (-59% and -63% respectively, p<0.001), IL-17A (-51% and -52% respectively, p<0.05) and IL-1β (-87% in both ice- and cold gas spray-treated rats, p<0.0001) gene expression. By contrast, TNF-α levels were unaffected by cryotherapy whatever the modality (Fig 4E to 4L).

### Effects of local cryotherapy on cytokine protein expression in AIA hind paws

**Fig 5:**
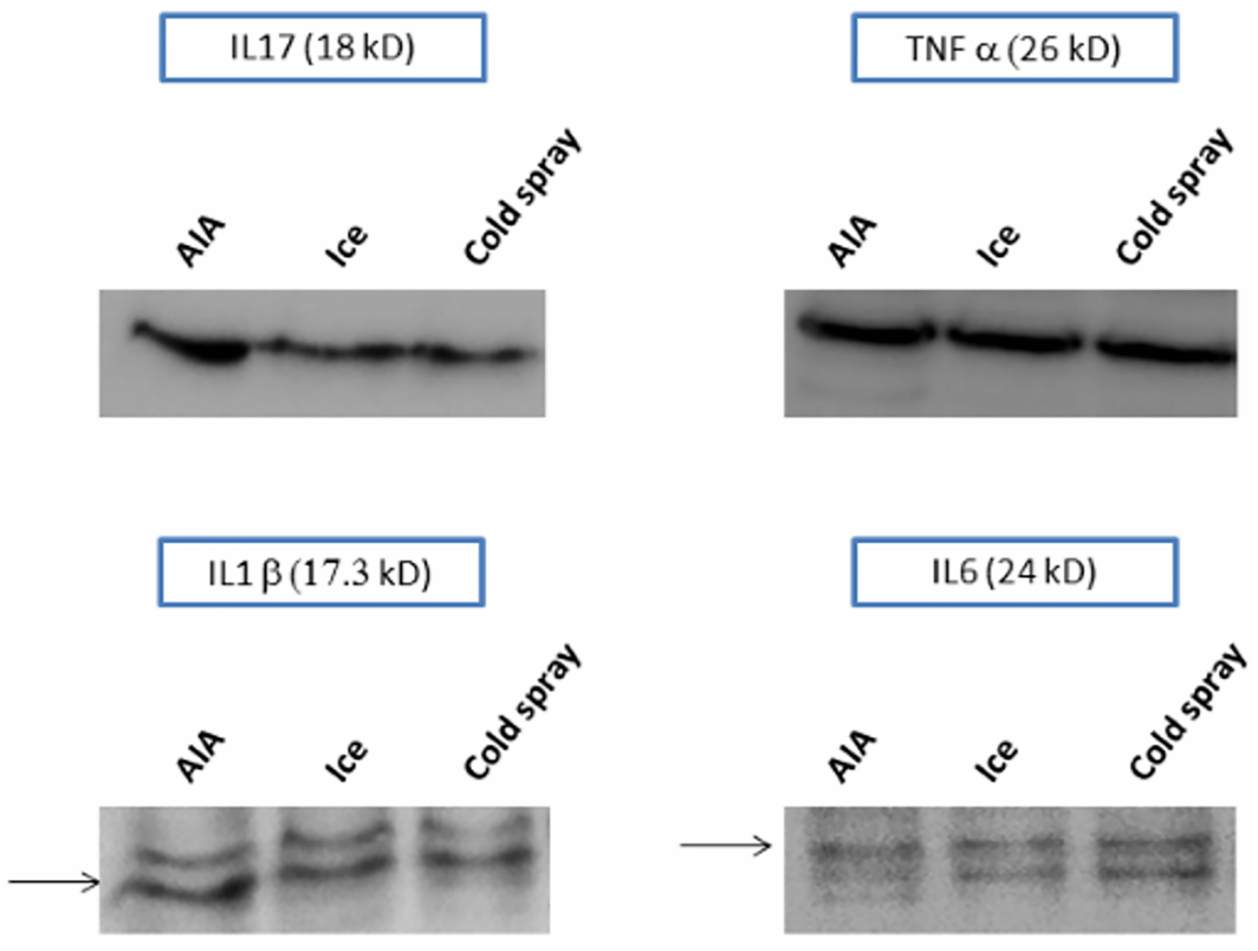
Effects of local cryotherapy on local cytokine protein expression (Western Blot on grinded hind paws). AIA rats were treated or not (control) either by being placed into cages lined with ice pops for 30 minutes (n = 10) or by intermittent cold gas spray applications on hind paws for 2 minutes (n = 9), twice a day for 14 consecutive days from day 11 to day 24 post-immunization. At day 24, pooled samples of grinded hind paw were prepared by mixing supernatants from the right and the left hind paws of each rat (n = 20 for non-treated controls and ice group; n = 18 for cold spray group), the same protein quantity being provided by each rat. Western blotting analyses of IL-17 (**A**), IL-6 (**B**), IL-1β (**C**) and TNF-α (**D**) protein expression were performed in these samples.

We evaluated the effects of local cryotherapy on cytokine protein expression in hind paws using Western Blot technique. By pooling the results from all the hind paws in each treatment group, we could show that both cryotherapy techniques markedly reduced the IL-17 protein expression and tended to reduce the IL-6 and IL-1β protein expression at day 24 in the treated hind paws compared to non-treated AIA hind paws. By contrast, local cryotherapy had no effect on the TNF-α protein expression (Fig 5).

### Effects of local cryotherapy on plasmatic cytokine levels in AIA

Then we determined whether the local effects of cryotherapy on cytokines were associated with systemic effects on cytokines.

**Fig 6:**
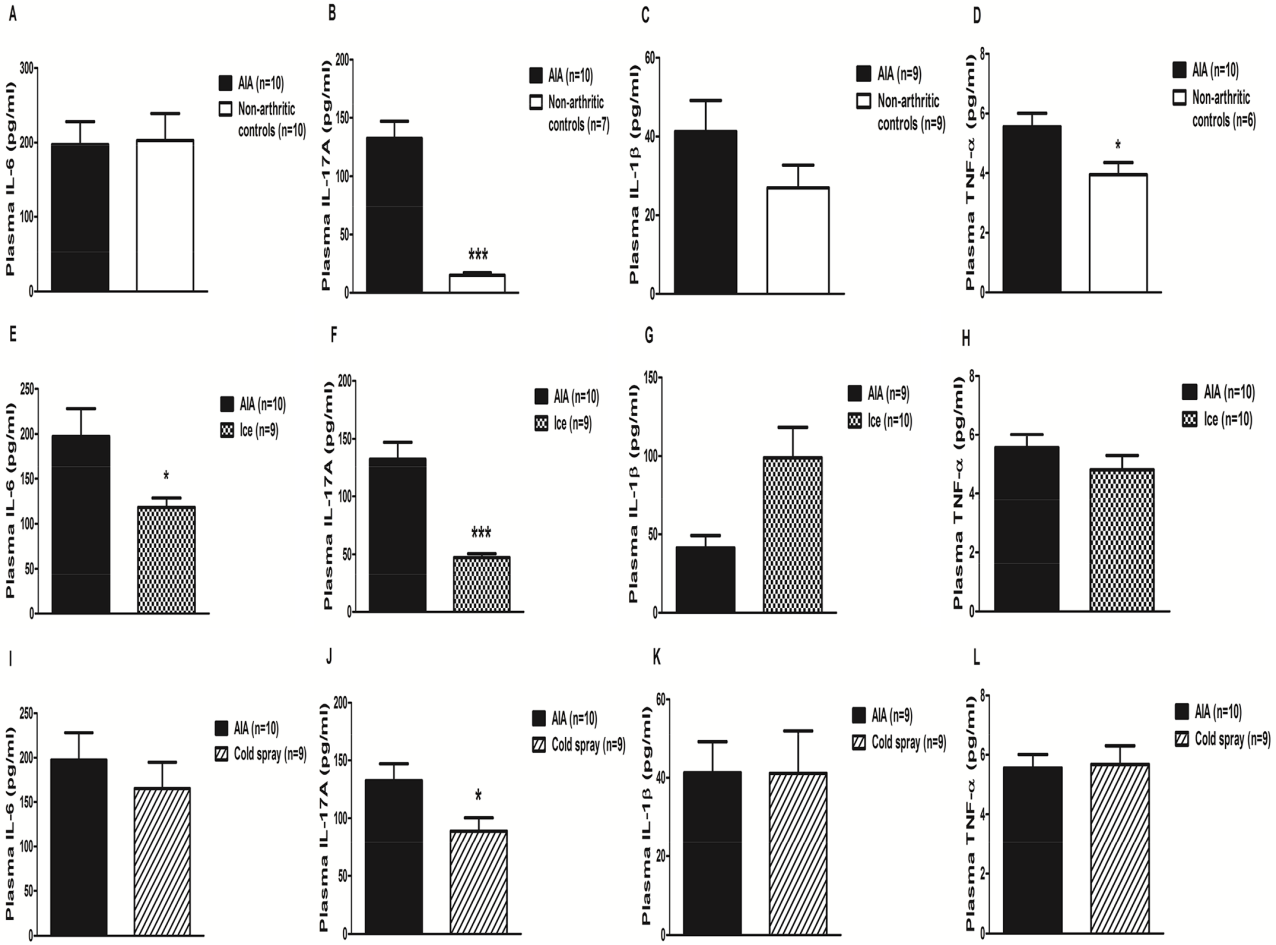
Cytokine plasmatic levels in non-arthritic, non-treated arthritic and cryotherapy-treated arthritic rats. Plasma levels of IL-6 (**A, E, I**), IL-17A (**B, F, J**), IL-1β (**C, G, K**), TNF-α (**D, H, L**) were assessed in AIA rats treated or not either by being placed into cages lined with ice pops for 30 minutes (n = 10) or by intermittent cold gas spray applications on hind paws for 2 minutes (n = 9), twice a day for 14 consecutive days from day 11 to day 24 post-immunization. Results are expressed as means ± SEM (n = 7–10 rats/group). Mann-Whitney tests were used. ***p<0.001, **p<0.1, *p<0.05.

Plasma IL-17A and TNF-α were significantly higher in AIA as compared to controls (p<0.001 –Fig 6B and p<0.05 –Fig 6D respectively). IL-1β tended to be higher in AIA but the difference did not reach significance (Fig 6C**)**. No change in IL-6 levels was observed (Fig 6A). As compared to AIA, ice-treated AIA had lower levels of IL-17A (p<10^−4^, Fig 6F), IL-6 (p = 0.05, Fig 6E) but unchanged TNF-α (Fig 6H) and IL-1β plasma levels (Fig 6G). Likewise, Cold-spray therapy significantly reduced IL-17A (p<0.05, Fig 6J) levels, with unchanged TNF-α (Fig 6L) and IL-1β levels (Fig 6K) but conversely to ice, it did not change IL-6 plasma levels (Fig 6I).

### Correlations between cytokines levels and clinical parameters

**Fig 7:**
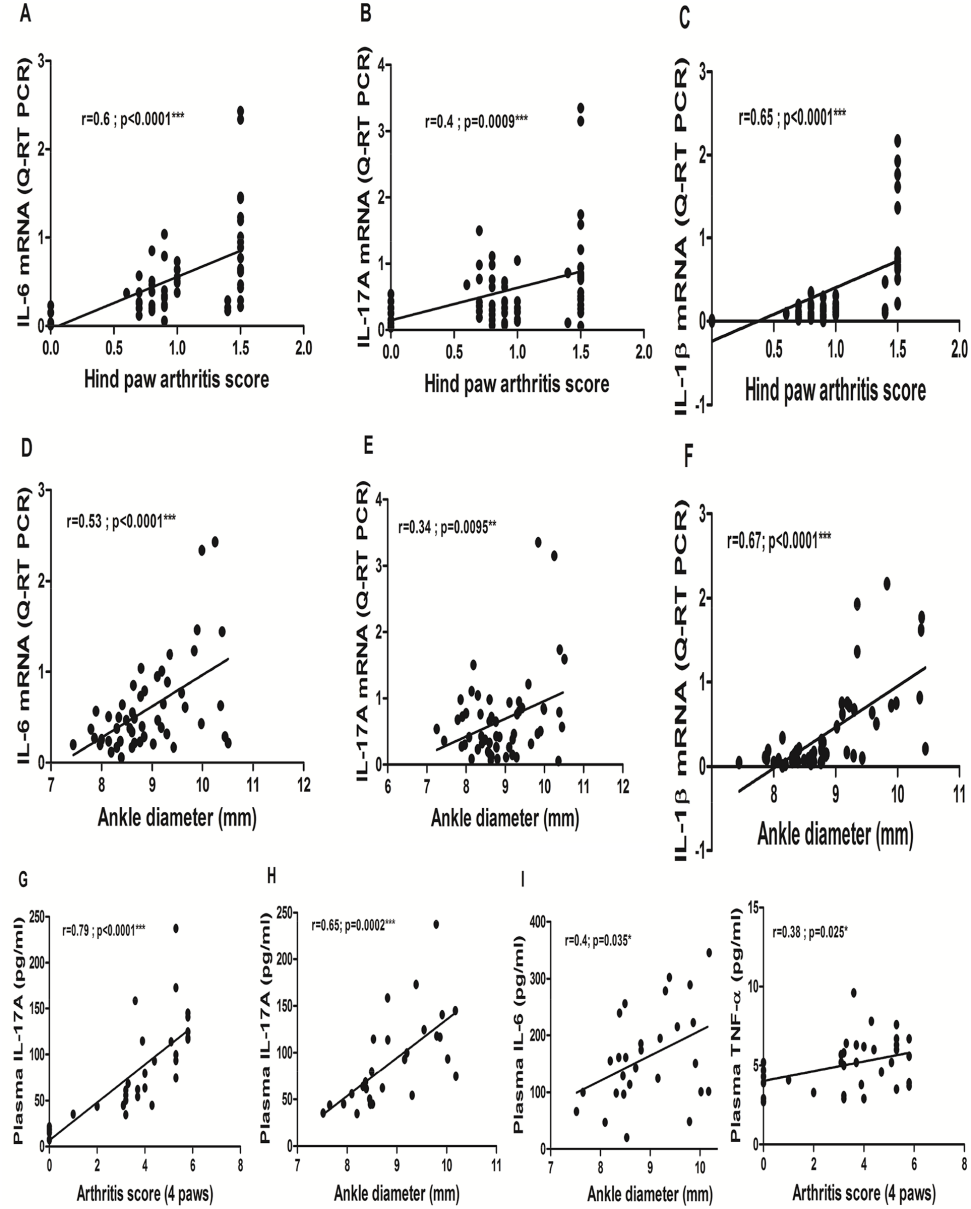
Correlations between pro-inflammatory cytokine levels and clinical parameters. Pearson’s r correlation coefficients were calculated between hind paw IL-6 (**A, D**), IL-17A (**B, E**), IL-β1 (**C, F**) gene expression levels and clinical parameters, and between plasma levels of IL-6 (**I**), IL-17A (**G, H**), TNF (**J**) and clinical parameters measured at day 24 post-immunization in AIA treated or not either by being placed into cages lined with ice pops for 30 minutes (n = 10) or by intermittent cold gas spray applications on hind paws for 2 minutes (n = 9), twice a day for 14 consecutive days from day 11 to day 24 post-immunization. For the arthritis score and ankle diameter, AIA rats from the 3 treatment groups and non-arthritic controls were considered (n = 60, n = 64, n = 60 pairs for hind paw IL-6, IL-17A and IL-1β mRNA levels and n = 35 pairs for both IL-17A and TNF-α plasma levels respectively). For ankle diameter, both hind paws from the 3 treatment groups were considered (n = 50, n = 56, n = 50 pairs for hind paw IL-6, IL-17A and IL-1β mRNA levels and n = 28 pairs for both IL-17A and IL-6 plasma levels respectively).

A significant correlation was observed between IL-6, IL-17A as well as IL-1β hind paw gene expression levels and the corresponding hind paws arthritis score (Fig 7A to 7C), and ankle diameter (Fig 7D to 7F). By contrast, no correlation was found between TNF-α gene expression levels and hind paw arthritis score (r = 0.17, p = 0.17 for arthritis score and r = -0.14, p = 0.29 for ankle diameter, not shown).

As regards plasma cytokines levels, plasma IL-17A levels correlated positively with the global arthritis score (r = 0.79 –p = 10^−4^ Fig 7G), with ankle diameter (r = 0.65 –p<0.001 –Fig 7H). Plasma IL-6 levels didn’t correlate with the arthritis score (r = -0.04, p = 0.83, not shown) but correlated with the ankle diameter (Fig 7I). TNF-α levels correlated with the arthritis score (Fig 7J) but not with ankle diameter (r = 0.22, p = 0.25, not shown). IL-1β levels didn’t correlate with the arthritis score (r = 0.14, p = 0.4) and quite surprisingly correlated negatively with the ankle diameter (r = -0.46, p = 0.015, not shown). Hind paw cytokine gene expression levels did not correlate with the corresponding protein plasma levels whatever the cytokine.

## Discussion

The seminal findings of this study are that 1) a sub-chronic treatment with local cryotherapy alone is efficient for reducing arthritis activity in AIA, 2) these beneficial effects are, at least in part, mediated by a down-regulation of joint and systemic IL-6/IL-17 pathway but independent on the TNFα pathway. These therapeutic and anti-inflammatory effects were recently partly confirmed in preliminary human studies [12,39,40].

Cryotherapy is a cheap, well-tolerated therapeutic option in an adjuvant setting in inflammatory rheumatic diseases. It is widely and empirically used in rheumatic diseases such as RA, with a paradoxically low level of evidence, mostly due to methodological issues and biases in clinical studies [12,41]. In murine models of arthritis, the therapeutic and anti-inflammatory effects of cryotherapy are also poorly documented. In AIA, WBC (liquid nitrogen at -110°C for 2 minutes, once a day for 2 weeks) applied in association with NSAIDs, corticosteroids and kinesitherapy reduced the number of swollen joints and increased histamine and cortisol in peripheral blood [42]. In another study, ice chips (0°C) applied 30 minutes a day for 10 days in rabbit zymosan-induced arthritis model tended to reduce synovial hyperplasia and cell infiltrate [43].

In the present study, we used the two most frequently used cryotherapy modalities: ice and to a lesser extent cold gas pulverization, which were not directly compared in the literature so far [12]. Ice (applied for 20–30 minutes) is supposed to provide a more in-deep and progressive cooling, while cold gas (applied for 2–5 minutes) induces a more ample temperature drop with associated vaso-active effects (a thermal shock with an intense vaso-constriction followed by a vaso-dilatation phenomenon). The optimal treatment periodicity and duration (twice a day for 14 days) was also suggested by the literature [12]. In other animal studies, ice chips applied for 30 minutes reduced intra-joint temperature to 17.1°C in zymosan-induced arthritis in rabbits [43] and ice induced a 16.6°C drop in healthy dog skin temperature, in line with our results in AIA [44]. In our study, ice was more precociously efficient on clinical arthritis and reduced the mean arthritis score and ankle diameter throughout the 14 days of treatment (2-way ANOVA), which was not the case for cold gas spray application, which showed a biphasic therapeutic response profile (Fig 3). We also observed erythema and local depilation on the cold gas-treated hind paws and one rat died from cardiac arrest after the first cold spray application. Thus, from a clinical point of view, local ice appeared to be more efficient and better tolerated than the cold gas pulverization technique we used in this model. As we found no cold gas devices dedicated to small animals, we used cold gas sprays conceived for human sportsmen and had to pulverize them intermittently on the hind paws as the cooling was very deep and brutal. However, even though cold gas showed a biphasic clinical response with an initial worsening of arthritis and was more poorly tolerated, it induced quite similar local and systemic anti-inflammatory effects than ice after 14 days of treatment. As both LC techniques are equally well-tolerated in arthritic patients [39], further research is now required in order to optimize cold gas application techniques in small rodents.

In the patella culture experiments, at day 24 after the induction of arthritis, IL-1β was the only up-regulated cytokine in the AIA patellae cultured at 37°C compared to non-arthritic controls, while the TNF-α protein was not detected (Fig 1). Lubberts et al. had already shown, in the same patella culture model, that the IL-1β protein levels were still up-regulated in the culture supernatants several days after the induction of arthritis (with concentration values in the same range as those we observed), while the TNF-α protein could only be detected a few hours after the induction, which could be an explanation for our results [28,45]. As concerns the IL-6 and IL-17A protein levels at 37°C, the cytokine concentrations tended to be higher in the patellar explants from arthritic rats compared to non-arthritic controls. In another article, in cultured patellae from mouse methylated bovine serum albumin induced arthritis, the authors reported high IL-17 protein levels in the supernatants 7 days after the induction of arthritis (while our evaluation was performed 24 days after the induction in our rat AIA model, at the end of the plateau phase) [30]. We found no data about the IL-6 protein levels obtained in this patella culture model. Importantly, data about cytokine protein levels in cultured patellae from non-arthritic rats or mice are not reported in the literature [28,30,45]. Furthermore, some authors previously reported in the literature that the cytokine protein level evaluation in hind paws and plasma in the monoarticular Wistar rat AIA model using Multiplex technique was more difficult and less sensitive than the corresponding gene transcription level measurement by Q-RT-PCR. Notably, the TNF-α protein levels were nearly undetectable in the synovial fluid from hind paws and in the plasma throughout the course of the arthritis, while the TNF-α gene transcription levels could only be measured in the early stages of arthritis [46]. This could explain the apparent lack of up-regulation of pro-inflammatory cytokine proteins in our AIA patella culture model, while in Q-RT-PCR experiments (Fig 4A to 4D), all the cytokine gene transcription levels were strongly up-regulated in the hind paws from non-treated arthritic rats compared to those from non-arthritic controls. The cytokine gene transcription levels at day 24 were probably a better reflection of the clinical arthritis, as they strongly correlated with the clinical parameters (except for TNF-α) in our AIA model (Fig 7). Furthermore, the Western Blot experiments in the hind paws at day 24 showed that TNF-α protein could be detected in the grinded hind paws. This could be explained by the fact that, in the AIA patella culture model, the cytokine protein levels were not directly measured in the hind paws, but in acellular supernatants from non-stimulated cultured patellae. In the Western Blot experiments, we could not perform any quantitative analysis of the cytokine protein expression in hind paws—by contrast with the Q-RT-PCR experiments—due to overall low cytokine protein expression levels. These difficulties in detecting cytokine protein levels in AIA might potentially be related to fast cytokine degradation processes by proteases. The TNF-α protein binding with the soluble TNF-α receptor might also interfere with the TNF-α protein detection by Multiplex kit antibodies.

Despite these relative difficulties to detect cytokine protein levels in cultured AIA patellar explants, we observed a strong inhibitory effect of hypothermia on IL-6 protein expression in the patella culture model. The effects of hypothermia (and cryotherapy) on cytokine levels had never been studied in murine models of arthritis so far. Quite surprisingly, punctual mild hypothermia (30°C for 2 hours) only down-regulated the IL-6 protein levels in patella culture supernatants, with no evident effect on the other pro-inflammatory cytokines. This result could however be explained, as in murine models of arthritis, IL-6 has been shown to induce subsequent Th-17 differenciation and then IL-17 secretion by Th-17 cells [4–6]. Therefore, as patellar explants were only cultured at 30°C for 2 hours, this duration was sufficient to decrease IL-6 protein levels but could have been too short to have any impact on the subsequent IL-17 secretion. We also observed that this punctual hypothermia had no impact on IL-1β protein levels, suggesting that a more prolonged or iterative cooling might be required in order to observe an effect on these cytokines. This hypothesis seems to be confirmed by the results of Q-RT-PCR and Western Blot experiments, which were performed in the hind paws after 14 days of LC (Figs 4E to 4L and 5). In summary, the results of this in vitro patella culture model suggest that punctual hypothermia might affect the IL-6 pathway more precociously than the other pro-inflammatory cytokines in the inflamed synovial tissue.

The anti-inflammatory effects of sub-chronic LC we observed *in vivo* in AIA appeared to be mostly IL-6/IL-17-driven. Indeed, both techniques reduced IL-6 and IL-17 gene and protein expression in treated hind paws and we observed significant correlations between gene expression and clinical parameters for these two cytokines (Fig 7). As suggested by the precocious decrease of IL-6 protein in our patella culture experiments, the effect of LC on synovial IL-6 pathway might have preceded and caused a subsequent decrease in IL-17A levels [4–6].

In the literature, the reported effects of hypothermia on the cytokine pathways are very diverse and sometimes contradictory depending on the considered models, irrespective of the hypothermia duration. Mild hypothermia previously showed inhibitory effects on the IL-6 pathway in human and mouse stroke, in HUVEC endothelial cell line culture, in rat pancreatitis and cardiac arrest models [20,47–49], which were at least partly mediated through NF-kB inhibition [20,47], and WBC also showed anti-IL-6 effects in RA patients without corticosteroids [50]. However, we found very few data about the effects of hypothermia on IL-17 levels: cultured peripheral T cells from healthy humans produced less IL-17 protein under hypothermic condition [51].

By contrast, both synovial mild hypothermia and sub-chronic LC had no effect on TNF-α gene and protein levels in our study. The effects of cryotherapy had never been studied in AIA so far. The reported effects of mild hypothermia in other (non-arthritic) murine models are conflicting [52–54]. In a model of rat pancreatitis, mild hypothermia applied for 3 hours reduced plasma IL-6 levels compared to normothermia [48]. In a femoral fracture model with hemorrhage in mice, mild hypothermia (3 hours) reduced plasma IL-6 but also TNF-α levels [53]. By contrast, in another hemorrhage model in rats, mild hypothermia (75 min) reduced plasma IL-6 with no effect on TNF-α [54]. This was also the case in a human glial cell line culture model [55].

So here we report for the first time therapeutic and anti-inflammatory effects of LC in AIA, which were mainly IL-6/IL-17-driven and TNF-α independent. The target cells would more likely be IL-6 secreting monocytes/macrophages, and then synovial and peripheral Th-17 T lymphocytes.

Another interesting result of our study is that even though cryotherapy was locally applied, it induced changes in systemic cytokine levels. Indeed, both LC techniques—while similarly repressing local IL-6, IL-17A and IL-1β gene expression and protein levels—also decreased the IL-17A plasma levels, while only ice reduced plasma IL-6 levels. The IL-6 protein levels also tended to decrease in the plasma of cold gas-treated AIA rats. We can hypothesize that ice induced a superior local and systemic anti-inflammatory effect in this model (as suggested by the evolution of the clinical parameters–Fig 3, which were significantly correlated with plasma IL-6 and IL-17A –Fig 7G to 7I). This could explain why the plasma IL-6 protein levels were only significantly reduced in ice-treated animals (Fig 6E and 6I) and plasma IL-17A decrease tended to be more ample in this group as well (Fig 6F and 6J). Globally, the systemic effect of both LC techniques appeared to be more pronounced on plasma IL-17A compared to IL-6 at day 24, also suggesting that the effects of LC on IL-6 might have preceded and potentially induced subsequent IL-17 inhibition [4–6]. Interestingly, plasma TNF-α protein levels correlated more weakly with the arthritis score at day 24 (Fig 7J), coherently with the other results reported above. In a study in human RA patients treated by local cold gas (twice a day for 10 days), the authors reported a decrease in TNF-α plasma levels with no effect on IL-6 plasma levels. However, the study was not controlled and the patients received concomitant systemic Disease Modifying Anti-inflammatory Drugs (DMARDs), NSAIDs, corticosteroids, and kinesitherapy, making it impossible to analyze the specific systemic anti-inflammatory effects of local cryotherapy by itself [17]. Notably, the concomitant systemic corticosteroids might have masked the effects of cryotherapy on plasma IL-6 protein levels, as previously suggested in another non-controlled study showing that WBC only decreased plasma IL-6 in RA patients without corticosteroids [50]. The effects of cryotherapy on IL-17A plasma levels had never been evaluated nor reported so far, to the best of our knowledge. In summary, our results suggest that LC applied sub-chronically, beyond its local anti-inflammatory effects in the joints, might also exert systemic anti-inflammatory effects, notably by down-regulating the IL-17/IL-6 pathway, a key actor in human RA pathogenesis.

The next step will be to try further elucidate the underlying molecular mechanisms. As no other data on the effects of LC in murine models of arthritis were published nor reported so far, we can only use indirect evidence from other models/medical fields and draw preliminary hypotheses in order to explain the clinical and biological effects we observed. First, hypothermia has been shown to inhibit numerous enzyme pathways, some of which may be critically involved in joint inflammation and destruction such as collagenases [18]. Furthermore, in human and murine models of brain ischaemia, mild tissue hypothermia was shown to inhibit the pro-inflammatory cytokine gene transcription through NF-kB pathway blockade [20]. Other pivotal intracellular protein kinase pathways might also be down-regulated under hypothermic conditions. In a human umbilical vein (HUVEC) cell line culture model, cells cultured under mild hypothermic condition (32°C versus 37°C) showed decreased IL-6 protein levels after 2 hours. However, IL-6 levels became significantly higher in this group after 6 hours, suggesting a potential duration threshold beyond which hypothermia might become pro-inflammatory. Interestingly, in these experiments, Ik-B (the natural inhibitor of the NF-kB pathway) levels were significantly increased after 20 minutes at 32°C and protein kinase ERK levels were down-regulated at 32°C versus 37°C after 20 and 60 minutes. Furthermore, COX-2 levels were down-regulated after 24 hours [47]. Furthermore, and quite similarly to the results we observed in LC-treated AIA rats, pharmacological selective COX-2 inhibitors significantly reduced the IL-6 gene transcription levels in the arthritic hind paws and plasma IL-6 levels with no effect on the TNF-α pathway in a monoarticular AIA model. By contrast, dexamethasone down-regulated both IL-6 and TNF-α pathways [32]. These two articles put together suggest that cryotherapy-induced mild hypothermia might down-regulate more specifically the IL-6 pathway through protein kinase and COX-2 blockade. We also observed an inhibitory effect of LC on IL-17 in AIA. A potential explanation could be suggested by experiments in cultured human RA synovial fibroblasts, showing that IL-6 contributed to an autocrine Th-17 induction. Furthermore, in these human RA fibroblast culture experiments, COX-2 inhibition by celecoxib down-regulated the TH-17 driven synovial inflammation. Therefore, beyond its repressive effects on the IL-6 pathway, LC might also have repressed the IL-17 pathway through COX-2 inhibition [56]. The potential impact of hypothermia on the JAK/STAT protein kinase pathway, a very promising therapeutic target in rheumatoid arthritis, has not been evaluated so far. Whether cryotherapy and hypothermia may inhibit all these important enzyme pathways in AIA and RA is still unknown.

As for the systemic anti-inflammatory effects of LC we observed, they might be partly mediated through autonomic nervous system involvement. Indeed, ice-water swimming was shown to increase peripheral norepinephrine levels in healthy subjects [16]. Furthermore, the α/β2-adrenergic receptor balance in the immune system has been shown to influence local and systemic inflammation as well as Th-1/Th-2 balance in AIA [57]. These systemic anti-inflammatory effects might also be partly related to NF-kB pathway inhibition in the spinal cord, which showed anti-inflammatory properties in AIA [58]. The molecular mechanisms involved in these previously unknown anti-IL-6 and anti-IL-17 effects of sub-chronic local cryotherapy in the context of murine arthritis (NF-kB dependent cytokine gene transcription inhibition? Temperature-dependent down-regulation of key enzyme pathways such as COX-2?) require further investigations and additional studies on this topic which could help elucidate and optimize the anti-inflammatory effects of local cryotherapy in human arthritis as well.

## Conclusions

The present study demonstrated that two LC techniques applied for 14 consecutive days have therapeutic effects in AIA. Ice showed a better efficacy and tolerance profile than cold gas spray. These therapeutic effects were associated with local and systemic anti-inflammatory effects, mainly through IL-6/IL-17 pathway inhibition. Cryotherapy techniques and protocols should be further evaluated and standardized in order to determine whether long-term LC might be a safer alternative to NSAIDs and corticosteroids, or offer dose-sparing effects, especially for vulnerable or elderly patients [13].

## Supporting information

S1 Fig Effects of mild hypothermia on the cell viability of cultured AIA patellar explants. After sacrifice, both patellae of each rat were dissected. Before culture, cell viability was assessed by measuring ATPase activity using fluorescence technique (CellTiter-Glo^®^ Luminescent Test, Promega). Viability didn’t differ significantly between 30°C- and 37°C- cultured patellae groups, suggesting that 2 hour-mild hypothermia had no influence on patellar explant cell viability. Paired Wilcoxon tests were used. Results are expressed as means ± SEM.
(TIF)

S2 Fig Supplemental figure 2.tif Time-course of arthritis score on hind paws in ice- (A) and cold gas-treated (B) AIA rats compared to controls. Results are expressed as means ± SEM (n = 9–10 rats/group). 2-way ANOVAs with Bonferroni post-tests were used. ***p<0.001, **p<0.1, *p<0.05.
(TIF)

## Abbreviations

AIA: Adjuvant-induced arthritis
COX-2: Cyclo-oxygenase-2
DAS-28: Disease activity score (28 joints)
DMARDs: Disease Modifying Anti-inflammatory Drugs
HUVEC: Human umbilical vein endothelial cell
i-NOS: inducible-NO-synthase
LC: Local cryotherapy
NFkB: Nuclear factor kappa B
NSAIDs: Non-steroidal anti-inflammatory drugs
PGE2: Prostaglandin E2
RA: Rheumatoid arthritis
Th-1: T helper-1
Treg: regulatory T cell
WBC: Whole-body cryotherapy

## References

[1] de Punder YMR, van Riel PLCM. Rheumatoid arthritis: understanding joint damage and physical disability in RA. Nat Rev Rheumatol. 2011;7: 260–261. doi: 10.1038/nrrheum.2011.49 21532640 10.1038/nrrheum.2011.49

[2] Brennan FM, McInnes IB. Evidence that cytokines play a role in rheumatoid arthritis. J Clin Invest. 2008;118: 3537–3545. doi: 10.1172/JCI36389 18982160 10.1172/JCI36389 PMC2575731

[3] Wright HL, Thomas HB, Moots RJ, Edwards SW. Interferon gene expression signature in rheumatoid arthritis neutrophils correlates with a good response to TNFi therapy. Rheumatol Oxf Engl. 2015;54: 188–193. doi: 10.1093/rheumatology/keu299 25125592 10.1093/rheumatology/keu299

[4] Kimura A, Kishimoto T. IL-6: regulator of Treg/Th17 balance. Eur J Immunol. 2010;40: 1830–1835. doi: 10.1002/eji.201040391 20583029 10.1002/eji.201040391

[5] Bettelli E, Korn T, Oukka M, Kuchroo VK. Induction and effector functions of T(H)17 cells. Nature. 2008;453: 1051–1057. doi: 10.1038/nature07036 18563156 10.1038/nature07036 PMC6280661

[6] Bettelli E, Carrier Y, Gao W, Korn T, Strom TB, Oukka M, et al Reciprocal developmental pathways for the generation of pathogenic effector TH17 and regulatory T cells. Nature. 2006;441: 235–238. doi: 10.1038/nature04753 16648838 10.1038/nature04753

[7] Lubberts E. Th17 cytokines and arthritis. Semin Immunopathol. 2010;32: 43–53. doi: 10.1007/s00281-009-0189-9 20127485 10.1007/s00281-009-0189-9 PMC2836464

[8] van Hamburg JP, Asmawidjaja PS, Davelaar N, Mus AMC, Cornelissen F, van Leeuwen JPTM, et al TNF blockade requires 1,25(OH)2D3 to control human Th17-mediated synovial inflammation. Ann Rheum Dis. 2012;71: 606–612. doi: 10.1136/annrheumdis-2011-200424 22219138 10.1136/annrheumdis-2011-200424

[9] Lubberts E. The IL-23-IL-17 axis in inflammatory arthritis. Nat Rev Rheumatol. 2015;11: 415–429. doi: 10.1038/nrrheum.2015.53 25907700 10.1038/nrrheum.2015.53

[10] Huizinga TWJ, Conaghan PG, Martin-Mola E, Schett G, Amital H, Xavier RM, et al Clinical and radiographic outcomes at 2 years and the effect of tocilizumab discontinuation following sustained remission in the second and third year of the ACT-RAY study. Ann Rheum Dis. 2015;74: 35–43. doi: 10.1136/annrheumdis-2014-205752 25169728 10.1136/annrheumdis-2014-205752 PMC4283697

[11] Zampeli E, Protogerou A, Stamatelopoulos K, Fragiadaki K, Katsiari CG, Kyrkou K, et al Predictors of new atherosclerotic carotid plaque development in patients with rheumatoid arthritis: a longitudinal study. Arthritis Res Ther. 2012;14: R44 doi: 10.1186/ar3757 22390577 10.1186/ar3757 PMC3446411

[12] Guillot X, Tordi N, Mourot L, Demougeot C, Dugué B, Prati C, et al Cryotherapy in inflammatory rheumatic diseases: a systematic review. Expert Rev Clin Immunol. 2014;10: 281–294. doi: 10.1586/1744666X.2014.870036 24345205 10.1586/1744666X.2014.870036

[13] Chatap G, De Sousa A, Giraud K, Vincent J-P, Acute Pain in the Elderly Study Group. Pain in the elderly: Prospective study of hyperbaric CO2 cryotherapy (neurocryostimulation). Jt Bone Spine Rev Rhum. 2007;74: 617–621. doi: 10.1016/j.jbspin.2006.10.011 17897861 10.1016/j.jbspin.2006.10.011

[14] Banfi G, Lombardi G, Colombini A, Melegati G. Whole-body cryotherapy in athletes. Sports Med Auckl NZ. 2010;40: 509–517. doi: 10.2165/11531940-000000000-00000 20524715 10.2165/11531940-000000000-00000

[15] Pournot H, Bieuzen F, Louis J, Mounier R, Fillard J-R, Barbiche E, et al Time-course of changes in inflammatory response after whole-body cryotherapy multi exposures following severe exercise. PloS One. 2011;6: e22748 doi: 10.1371/journal.pone.0022748 21829501 10.1371/journal.pone.0022748 PMC3145670

[16] Leppäluoto J, Westerlund T, Huttunen P, Oksa J, Smolander J, Dugué B, et al Effects of long-term whole-body cold exposures on plasma concentrations of ACTH, beta-endorphin, cortisol, catecholamines and cytokines in healthy females. Scand J Clin Lab Invest. 2008;68: 145–153. doi: 10.1080/00365510701516350 18382932 10.1080/00365510701516350

[17] Jastrząbek R, Straburzyńska-Lupa A, Rutkowski R, Romanowski W. Effects of different local cryotherapies on systemic levels of TNF-α, IL-6, and clinical parameters in active rheumatoid arthritis. Rheumatol Int. 2013;33: 2053–2060. doi: 10.1007/s00296-013-2692-5 23397259 10.1007/s00296-013-2692-5

[18] Harris ED, McCroskery PA. The influence of temperature and fibril stability on degradation of cartilage collagen by rheumatoid synovial collagenase. N Engl J Med. 1974;290: 1–6. doi: 10.1056/NEJM197401032900101 4357162 10.1056/NEJM197401032900101

[19] Dugué B, Smolander J, Westerlund T, Oksa J, Nieminen R, Moilanen E, et al Acute and long-term effects of winter swimming and whole-body cryotherapy on plasma antioxidative capacity in healthy women. Scand J Clin Lab Invest. 2005;65: 395–402. doi: 10.1080/00365510510025728 16081362 10.1080/00365510510025728

[20] Yenari MA, Han HS. Influence of hypothermia on post-ischemic inflammation: role of nuclear factor kappa B (NFkappaB). Neurochem Int. 2006;49: 164–169. doi: 10.1016/j.neuint.2006.03.016 16750872 10.1016/j.neuint.2006.03.016

[21] Truettner JS, Alonso OF, Dalton Dietrich W. Influence of therapeutic hypothermia on matrix metalloproteinase activity after traumatic brain injury in rats. J Cereb Blood Flow Metab Off J Int Soc Cereb Blood Flow Metab. 2005;25: 1505–1516. doi: 10.1038/sj.jcbfm.9600150 15959464 10.1038/sj.jcbfm.9600150

[22] Oliver SJ, Brahn E. Combination therapy in rheumatoid arthritis: the animal model perspective. J Rheumatol Suppl. 1996;44: 56–60. 8833054

[23] Bolon B, Stolina M, King C, Middleton S, Gasser J, Zack D, et al Rodent preclinical models for developing novel antiarthritic molecules: comparative biology and preferred methods for evaluating efficacy. J Biomed Biotechnol. 2011;2011: 569068 doi: 10.1155/2011/569068 21253435 10.1155/2011/569068 PMC3022224

[24] Sakaguchi N, Takahashi T, Hata H, Nomura T, Tagami T, Yamazaki S, et al Altered thymic T-cell selection due to a mutation of the ZAP-70 gene causes autoimmune arthritis in mice. Nature. 2003;426: 454–460. doi: 10.1038/nature02119 14647385 10.1038/nature02119

[25] Lloyd M., Wolfensohn S. Practical use of distress scoring systems in the application of humane end points. Humane Endpoints in Animal.

[26] Snekhalatha U, Anburajan M, Venkatraman B, Menaka M. Evaluation of complete Freund’s adjuvant-induced arthritis in a Wistar rat model. Comparison of thermography and histopathology. Z Rheumatol. 2013;72: 375–382. doi: 10.1007/s00393-012-1083-8 23208192 10.1007/s00393-012-1083-8

[27] Van den Berg WK, Van den Putte L. The mouse patella assay: an easy method of quantitating articular chondrocyte function in vivo and in vitro. Rheumatol Int. 1982;1: 165–9.

[28] Lubberts E, Joosten LA, Helsen MM, van den Berg WB. Regulatory role of interleukin 10 in joint inflammation and cartilage destruction in murine streptococcal cell wall (SCW) arthritis. More therapeutic benefit with IL-4/IL-10 combination therapy than with IL-10 treatment alone. Cytokine. 1998;10: 361–369. doi: 10.1006/cyto.1997.0298 9619374 10.1006/cyto.1997.0298

[29] Lubberts E, Joosten LA, van de Loo FA, van den Gersselaar LA, van den Berg WB. Reduction of interleukin-17-induced inhibition of chondrocyte proteoglycan synthesis in intact murine articular cartilage by interleukin-4. Arthritis Rheum. 2000;43: 1300–1306. doi: 10.1002/1529-0131(200006)43:6<1300::AID-ANR12>3.0.CO;2-D 10857788 10.1002/1529-0131(200006)43:6<1300::AID-ANR12>3.0.CO;2-D

[30] van Hamburg JP, Mus A-M, de Bruijn MJW, de Vogel L, Boon L, Cornelissen F, et al GATA-3 protects against severe joint inflammation and bone erosion and reduces differentiation of Th17 cells during experimental arthritis. Arthritis Rheum. 2009;60: 750–759. doi: 10.1002/art.24329 19248112 10.1002/art.24329

[31] Oosterveld FG, Rasker JJ. Effects of local heat and cold treatment on surface and articular temperature of arthritic knees. Arthritis Rheum. 1994;37: 1578–1582. 7980668 10.1002/art.1780371104

[32] Anderson GD, Hauser SD, McGarity KL, Bremer ME, Isakson PC, Gregory SA. Selective inhibition of cyclooxygenase (COX)-2 reverses inflammation and expression of COX-2 and interleukin 6 in rat adjuvant arthritis. J Clin Invest. 1996;97: 2672–2679. doi: 10.1172/JCI118717 8647962 10.1172/JCI118717 PMC507355

[33] Earp JC, Dubois DC, Molano DS, Pyszczynski NA, Keller CE, Almon RR, et al Modeling corticosteroid effects in a rat model of rheumatoid arthritis I: mechanistic disease progression model for the time course of collagen-induced arthritis in Lewis rats. J Pharmacol Exp Ther. 2008;326: 532–545. doi: 10.1124/jpet.108.137372 18448865 10.1124/jpet.108.137372 PMC2574807

[34] Schmidt-Weber CB, Pohlers D, Siegling A, Schädlich H, Buchner E, Volk HD, et al Cytokine gene activation in synovial membrane, regional lymph nodes, and spleen during the course of rat adjuvant arthritis. Cell Immunol. 1999;195: 53–65. doi: 10.1006/cimm.1999.1509 10433797 10.1006/cimm.1999.1509

[35] Ayer LM, Issekutz AC, Waterhouse CC, Stadnyk AW. Cytokine mRNA in the joints and draining lymph nodes of rats with adjuvant arthritis and effects of cyclosporin A. Inflammation. 2000;24: 447–461. 10921508 10.1023/a:1007064212462

[36] Hu W, Xia L-J, Chen F-H, Wu F-R, Tang J, Chen C-Z, et al Recombinant human endostatin inhibits adjuvant arthritis by down-regulating VEGF expression and suppression of TNF-α, IL-1β production. Inflamm Res Off J Eur Histamine Res Soc Al. 2012;61: 827–835. doi: 10.1007/s00011-012-0477-z 22610149 10.1007/s00011-012-0477-z

[37] Rioja I, Bush KA, Buckton JB, Dickson MC, Life PF. Joint cytokine quantification in two rodent arthritis models: kinetics of expression, correlation of mRNA and protein levels and response to prednisolone treatment. Clin Exp Immunol. 2004;137: 65–73. doi: 10.1111/j.1365-2249.2004.02499.x 15196245 10.1111/j.1365-2249.2004.02499.x PMC1809073

[38] Portanova JP, Zhang Y, Anderson GD, Hauser SD, Masferrer JL, Seibert K, et al Selective neutralization of prostaglandin E2 blocks inflammation, hyperalgesia, and interleukin 6 production in vivo. J Exp Med. 1996;184: 883–891. 9064348 10.1084/jem.184.3.883 PMC2192784

[39] Guillot X, Tordi N, Prati C, Verhoeven F, Pazart L, Wendling D. Cryotherapy decreases synovial Doppler activity and pain in knee arthritis: A randomized-controlled trial. Jt Bone Spine Rev Rhum. 2016; doi: 10.1016/j.jbspin.2016.09.004 27825572 10.1016/j.jbspin.2016.09.004

[40] Guillot X, Tordi N, Laheurte C, Pazart L, Prati C, Saas P, et al [SAT0626] Local cryotherapy (pulsed CO2 or ice) decreases IL-6, IL-1β and VEGF synovial levels in knee arthritis. Ann Rheum Dis. 2016;75:Suppl2: 896 doi: 10.1136/annrheumdis-2016-eular.1699

[41] Welch V, Brosseau L, Shea B, McGowan J, Wells G, Tugwell P. Thermotherapy for treating rheumatoid arthritis. Cochrane Database Syst Rev. 2001; CD002826. doi: 10.1002/14651858.CD002826 10.1002/14651858.CD002826 11406046

[42] Wojtecka-Lukasic E, Ksiezopolska-Orlowska K, Burakowski T, Marton A, Maoelinska D, Maoelinski W, et al Effect of cryotherapy on adjuvant arthritis in the rat. Ann Rheum Dis. 2002;61 Suppl.

[43] Weinberger A, Giler S. Treatment of inflammatory synovitis with ice application. Arthritis Rheum. 1995;38 Suppl: S242.

[44] Wakim KG, Porter AN, Krusen FH. Influence of physical agents and of certain drugs on intra-articular temperature. Arch Phys Med Rehabil. 1951;32: 714–721. 14886159

[45] Lubberts E, Joosten LA, Oppers B, van den Bersselaar L, Coenen-de Roo CJ, Kolls JK, et al IL-1-independent role of IL-17 in synovial inflammation and joint destruction during collagen-induced arthritis. J Immunol Baltim Md 1950. 2001;167: 1004–1013. 10.4049/jimmunol.167.2.1004 11441109

[46] Paquet J, Goebel J-C, Delaunay C, Pinzano A, Grossin L, Cournil-Henrionnet C, et al Cytokines profiling by multiplex analysis in experimental arthritis: which pathophysiological relevance for articular versus systemic mediators? Arthritis Res Ther. 2012;14: R60 doi: 10.1186/ar3774 22414623 10.1186/ar3774 PMC3446427

[47] Diestel A, Roessler J, Berger F, Schmitt KRL. Hypothermia downregulates inflammation but enhances IL-6 secretion by stimulated endothelial cells. Cryobiology. 2008;57: 216–222. doi: 10.1016/j.cryobiol.2008.08.005 18790695 10.1016/j.cryobiol.2008.08.005

[48] Fujimoto K, Fujita M, Tsuruta R, Tanaka R, Shinagawa H, Izumi T, et al Early induction of moderate hypothermia suppresses systemic inflammatory cytokines and intracellular adhesion molecule-1 in rats with caerulein-induced pancreatitis and endotoxemia. Pancreas. 2008;37: 176–181. doi: 10.1097/MPA.0b013e318162cb26 18665080 10.1097/MPA.0b013e318162cb26

[49] Tortorici MA, Mu Y, Kochanek PM, Xie W, Poloyac SM. Moderate hypothermia prevents cardiac arrest-mediated suppression of drug metabolism and induction of interleukin-6 in rats. Crit Care Med. 2009;37: 263–269. doi: 10.1097/CCM.0b013e3181931ed3 19050605 10.1097/CCM.0b013e3181931ed3 PMC2613167

[50] Straub RH, Pongratz G, Hirvonen H, Pohjolainen T, Mikkelsson M, Leirisalo-Repo M. Acute cold stress in rheumatoid arthritis inadequately activates stress responses and induces an increase of interleukin 6. Ann Rheum Dis. 2009;68: 572–578. doi: 10.1136/ard.2008.089458 18413439 10.1136/ard.2008.089458

[51] Matsui T, Kawahara N, Kimoto A, Yoshida Y. Hypothermia Reduces but Hyperthermia Augments T Cell-Derived Release of Interleukin-17 and Granzyme B that Mediate Neuronal Cell Death. Neurocrit Care. 2015;23: 116–126. doi: 10.1007/s12028-014-0094-5 25479711 10.1007/s12028-014-0094-5

[52] Webster CM, Kelly S, Koike MA, Chock VY, Giffard RG, Yenari MA. Inflammation and NFkappaB activation is decreased by hypothermia following global cerebral ischemia. Neurobiol Dis. 2009;33: 301–312. doi: 10.1016/j.nbd.2008.11.001 19063968 10.1016/j.nbd.2008.11.001 PMC2737398

[53] Hildebrand F, van Griensven M, Giannoudis P, Luerig A, Harwood P, Harms O, et al Effects of hypothermia and re-warming on the inflammatory response in a murine multiple hit model of trauma. Cytokine. 2005;31: 382–393. doi: 10.1016/j.cyto.2005.06.008 16099670 10.1016/j.cyto.2005.06.008

[54] Gundersen Y, Vaagenes P, Pharo A, Valø ET, Opstad PK. Moderate hypothermia blunts the inflammatory response and reduces organ injury after acute haemorrhage. Acta Anaesthesiol Scand. 2001;45: 994–1001. 11576051 10.1034/j.1399-6576.2001.450812.x

[55] Gibbons H, Sato TA, Dragunow M. Hypothermia suppresses inducible nitric oxide synthase and stimulates cyclooxygenase-2 in lipopolysaccharide stimulated BV-2 cells. Brain Res Mol Brain Res. 2003;110: 63–75. 12573534 10.1016/s0169-328x(02)00585-5

[56] Paulissen SMJ, van Hamburg JP, Davelaar N, Asmawidjaja PS, Hazes JMW, Lubberts E. Synovial fibroblasts directly induce Th17 pathogenicity via the cyclooxygenase/prostaglandin E2 pathway, independent of IL-23. J Immunol Baltim Md 1950. 2013;191: 1364–1372. doi: 10.4049/jimmunol.1300274 23817417 10.4049/jimmunol.1300274

[57] Lubahn CL, Lorton D, Schaller JA, Sweeney SJ, Bellinger DL. Targeting α- and β-Adrenergic Receptors Differentially Shifts Th1, Th2, and Inflammatory Cytokine Profiles in Immune Organs to Attenuate Adjuvant Arthritis. Front Immunol. 2014;5: 346 doi: 10.3389/fimmu.2014.00346 25157248 10.3389/fimmu.2014.00346 PMC4127464

[58] Luo J-G, Zhao X-L, Xu W-C, Zhao X-J, Wang J-N, Lin X-W, et al Activation of spinal NF-κB/p65 contributes to peripheral inflammation and hyperalgesia in rat adjuvant-induced arthritis. Arthritis Rheumatol Hoboken NJ. 2014;66: 896–906. doi: 10.1002/art.38328 24757142 10.1002/art.38328

