# Supplementary figures and images for: Local cryotherapy improves adjuvant-induced arthritis through down-regulation of IL-6 / IL-17 pathway but independently of TNFα
Source: PLoS One. 2017 Jul 31;12(7):e0178668. doi: 10.1371/journal.pone.0178668 (PMC5536266; doi:10.1371/journal.pone.0178668)

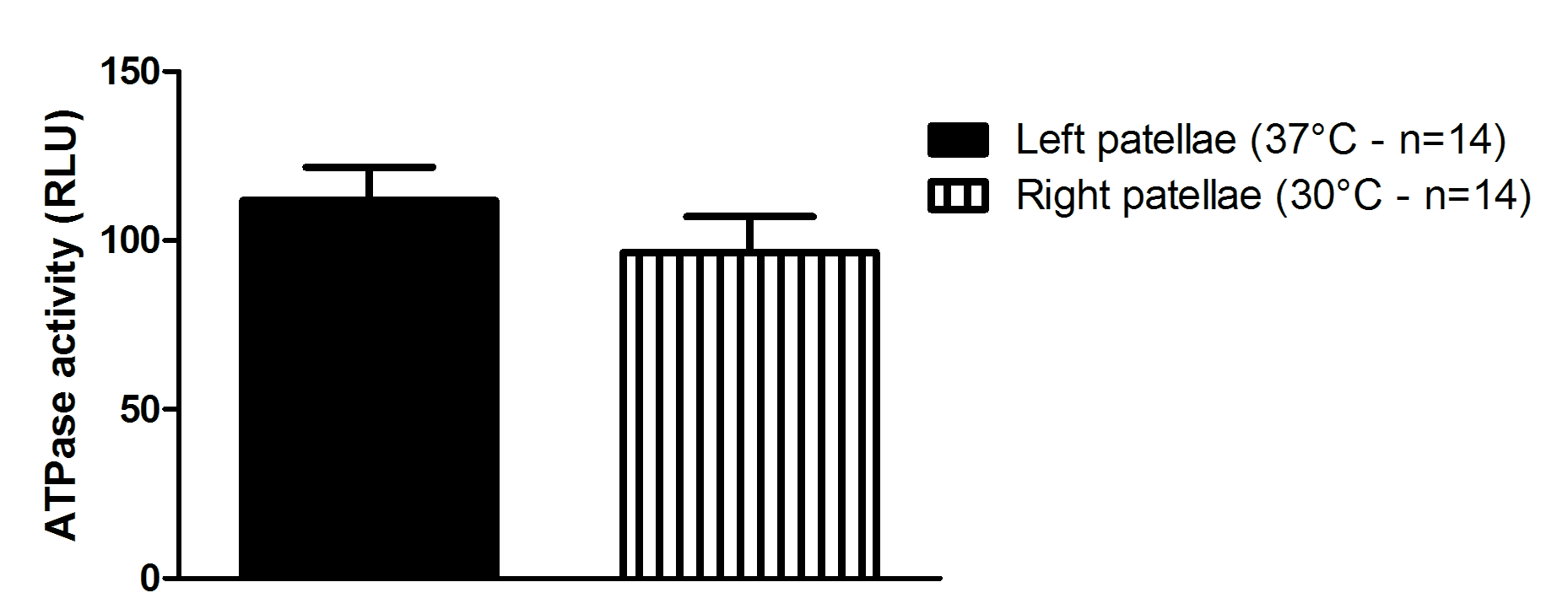

Supplement: S1 Fig — After sacrifice, both patellae of each rat were dissected. Before culture, cell viability was assessed by measuring ATPase activity using fluorescence technique (CellTiter-Glo® Luminescent Test, Promega). Viability didn’t differ significantly between 30°C- and 37°C- cultured patellae groups, suggesting that 2 hour-mild hypothermia had no influence on patellar explant cell viability. Paired Wilcoxon tests were used. Results are expressed as means ± SEM. (TIF) [file pone.0178668.s001.tif]

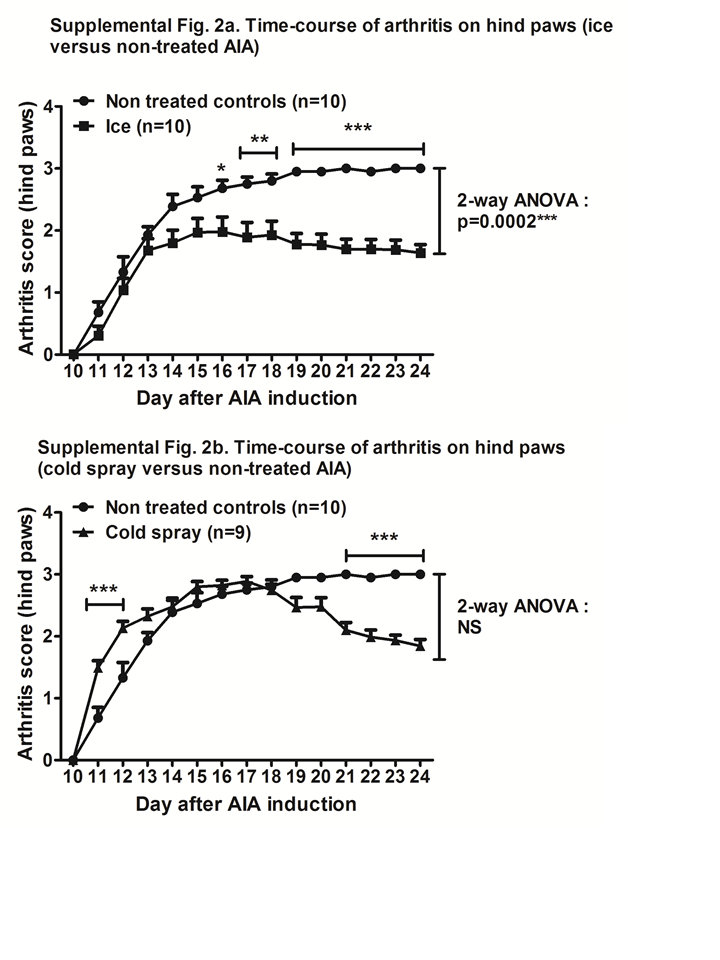

Supplement: S2 Fig — Results are expressed as means ± SEM (n = 9–10 rats/group). 2-way ANOVAs with Bonferroni post-tests were used. ***p<0.001, **p<0.1, *p<0.05. (TIF) [file pone.0178668.s002.tif]
